# Adverse Selection in Community Based Health Insurance among Informal Workers in Bangladesh: An EQ-5D Assessment

**DOI:** 10.3390/ijerph15020242

**Published:** 2018-01-31

**Authors:** Sayem Ahmed, Abdur Razzaque Sarker, Marufa Sultana, Sanchita Chakrovorty, Md. Zahid Hasan, Andrew J. Mirelman, Jahangir A. M. Khan

**Affiliations:** 1 Health Economics and Financing Research Group, Health Systems and Population Studies Division, Bangladesh (icddr,b), Dhaka 1212, Bangladesh; arazzaque@icddrb.org (A.R.S.); marufa@icddrb.org (M.S.); scakrov@purdue.edu (S.C.); md.zahid@icddrb.org (M.Z.H.); Jahangir.Khan@lstmed.ac.uk (J.A.M.K.); 2 Health Economics and Policy Research Group, Department of Learning, Informatics, Management and Ethics (LIME), Karolinska Institutet, SE-171 77 Stockholm, Sweden; 3 Department of Management Science, University of Strathclyde, Glasgow G1 1XQ, UK; 4 Faculty of Health, Deakin University, Melbourne, VIC 3125, Australia; 5 Department of Agriculture Economics, Purdue University, IN 47907, USA; 6 Centre for Health Economics, University of York, York YO10 5DD, UK; andrew.mirelman@york.ac.uk; 7 Department of Clinical Sciences, Liverpool School of Tropical Medicine, Liverpool L3 5QA, UK

**Keywords:** adverse selection, community based health insurance, EQ-5D

## Abstract

Community-based Health Insurance (CBHI) schemes are recommended for providing financial risk protection to low-income informal workers in Bangladesh. We assessed the problem of adverse selection in a pilot CBHI scheme in this context. In total, 1292 (646 insured and 646 uninsured) respondents were surveyed using the Bengali version of the EuroQuol-5 dimensions (EQ-5D) questionnaire for assessing their health status. The EQ-5D scores were estimated using available regional tariffs. Multiple logistic regression was applied for predicting the association between health status and CBHI scheme enrolment. A higher number of insured reported problems in mobility (7.3%; *p* = 0.002); self-care (7.1%; *p* = 0.000) and pain and discomfort (7.7%; *p* = 0.005) than uninsured. The average EQ-5D score was significantly lower among the insured (0.704) compared to the uninsured (0.749). The regression analysis showed that those who had a problem in mobility (OR = 1.65; 95% CI: 1.25–2.17); self-care (OR = 2.29; 95% CI: 1.62–3.25) and pain and discomfort (OR = 1.43; 95% CI: 1.13–1.81) were more likely to join the scheme. Individuals with higher EQ-5D scores (OR = 0.46; 95% CI: 0.31–0.69) were less likely to enroll in the scheme. Given that adverse selection was evident in the pilot CBHI scheme, there should be consideration of this problem when planning scale-up of these kind of schemes.

## 1. Introduction

Out-of-pocket (OOP) healthcare payment is the predominant financing mechanism in many low- and middle-income countries (LMICs) [1]. It is a source of 48% of total healthcare expenditure in low-income countries [2]. This high reliance on OOP healthcare payment results in catastrophic healthcare expenditure and impoverishment in LMICs [2]. The World Health Organization suggested increasing dependency on innovative prepayment healthcare financing mechanisms with the provision of risk pooling for mitigating this problem [3]. Considering the limited revenue generated through taxation in low- and middle-income countries (LMICs), community-based health insurance (CBHI) schemes are a potential source of financing healthcare in these countries [1,4,5].

In Bangladesh, around 67% of the healthcare costs are borne by the OOP payments which result in high catastrophic health expenditures (16.5%) among the poor [6,7]. To reduce this burden, the government of Bangladesh adopted its first-ever healthcare financing strategy in 2012 [8]. In this strategy, CBHI schemes are recommended for providing financial risk protection for low-income informal workers in Bangladesh.

CBHI schemes have rapidly grown over the last two decades as a health financing tool in LMICs where other insurance (e.g., government financed) is absent or may not be feasible [9]. Development actors are increasingly considering CBHI as an instrument that can enable easy access to quality healthcare in an affordable way [10]. The ultimate aims of such schemes are to facilitate access to healthcare and increase financial protection against the huge burden of OOP expenditure [11].

In most of the CBHI schemes, there is a chance of information asymmetry between the insurer and the insured. In particular, potential members know their disease risk levels better than the insurers. Therefore, high-risk members are able to purchase insurance at a lower premium than their disease risk would imply since the premium is fixed based on the average disease risk of the potential members and willingness-to-pay which is known as adverse selection [12]. In CBHI schemes, membership is voluntary in nature. However, several studies reported that such membership can cause adverse selection in these schemes [9,13,14]. In the presence of adverse selection, the financial sustainability of such schemes become vulnerable [15].

A large body of literature addressed the problem of adverse selection in cases of employer-sponsored or publicly managed insurance schemes [9]. However, limited studies have analyzed such problems in CBHI schemes in LMICs. The evidence reported both types of results; absence and presence of adverse selection. A study in China reported the adverse selection in the Rural Mutual Health Care [14]. Noterman et al., 1995 found adverse selection among women in their reproductive age under a prepayment scheme with coverage for hospitalization [16]. Dror et al., 2005 reported the absence of adverse selection in the Micro Health Insurance Units in the Philippines by assessing morbidity between insured and uninsured [17]. A similar result was found in a study conducted by De Allegri et al., 2006 for the CBHI scheme in Burkina Faso. Parmar et al., 2012 reported of having week evidence of adverse selection in a CBHI scheme in rural Africa [9,18].

Since 2012, when the government of Bangladesh recommended CBHI schemes as a mechanism of healthcare financing for covering informal worker [8], there has been a growing interest in these types of schemes from policymakers, development partners, and health program implementers. In this context, it is important to analyze the presence of adverse selection to facilitate more effective health planning. In Bangladesh, there are no studies on whether adverse selection is a problem in CBHI schemes. We aim to examine this issue in a pilot CBHI scheme among the informal workers in Bangladesh.

## 2. Materials and Methods

### 2.1. CBHI Scheme

A CBHI scheme comprising a group of informal workers was implemented through forming a labor cooperative organization. The enrolment in the CBHI scheme was voluntary and the organization engaged in a number of marketing interventions (such as group meetings, and individual counseling by marketing staff) to enroll members in the scheme. Under the membership package for an informal worker, the other members of his/her household were considered as beneficiaries. A brief description of the CBHI scheme under this study is presented below.

Target population: Informal workers with low income and their household members in Chandpur sub-district (comprising urban and rural areas) of Bangladesh were the target population of this scheme.

Implementation entity: Labor Association for Social Protection, a cooperative organization, under the Ministry of Local Government and Rural Development was the main implementation entity.

Beneficiaries: Up to six members of each household were entitled to health benefits for one membership card. The children under 5 were automatically enrolled in the scheme and not counted under the beneficiary limit.

Benefits package: The benefits package includes both health (e.g., discounted consultation, medicine, diagnostic test, and hospitalization) and non-health benefits (e.g., savings opportunity and technical training). The benefits package of the scheme is presented in Table 1.

Premium: The premium was 600 BDT (7.72 USD) per household per year which is 2.68% of the informal worker annual income 22,352 BDT (287.60 USD) [20].

### 2.2. Study Design

A quasi-experimental approach was employed to examine the differences in health status among those who joined the CBHI scheme (insured) and those who did not (uninsured). The uninsured had similar observable characteristics through matching on age, gender, residence, and household composition (e.g., presence of a child, elderly, and number of reproductive-aged women).

### 2.3. Study Population and Sample

This study was conducted in *Chandpur Sadar* Upazila (sub-district). It consists of 9 Unions (areas under sub-district) and the CBHI scheme was offered in 7 of them. We performed a cross-sectional survey of 1292 households (646 insured and 646 uninsured) from these 7 Unions during April–June 2014.

### 2.4. Data Collection

The household members aged 18 and above with involvement in household economic activities and decision making were selected as respondents for this study considering their availability for interview. We explained the study objectives to the respondents before interviewing them. We only interviewed individuals who provided informed written consent.

The respondents were interviewed using a pre-tested structured questionnaire. Face-to-face interview with respondents were conducted using the Bengali version of the EuroQuol-5 dimensions (EQ-5D) instrument. In addition, we collected demographic characteristics of individual members and households.

### 2.5. Health Status Measurement

The health status of study participants was estimated using the EQ-5D instrument. EuroQol group, a European-based researchers, developed the instrument to estimate health status score based on the individual responses [21]. This instrument contains two measurement parts. The first part defines the self-reported general state of health with five dimensions (mobility, self-care, usual activities, pain/discomfort, and anxiety/depression) which is known as the descriptive part. Each of these dimensions has three levels (no problems, some problems, and severe problems). The second part contains a self-reported visual analog scale (VAS) scale of 20 cm length. The individuals are asked to mark anywhere on the scale based on how well they feel about their health state today. In order to obtain health state scores, the scale has endpoints of 100 and 0, where 100 indicates the “best imaginable health state” and 0 indicates the “worst imaginable health state” [22]. In this study, we used a Bengali version of the ED-5D instrument available for Bangladesh to estimate the individual’s health status.

The EQ-5D index tariff assigns a single index value for all hypothetical health states identified by the EQ-5D instrument. The assigned values are estimated using a scale of 0 to 1, where 0 represents dead and 1 represents perfect health. The values can be negative (up to −0.59) in cases of severely detrimental health conditions [23]. We used a Thailand based regional tariff in the absence of any such weights in Bangladesh context.

The individual response of having any problem (reported levels 2 and 3) in each of the EQ-5D dimension was calculated for the insured group and corresponding uninsured group. *t*-Tests were used to identify statistical significance of the difference in health state scores, demographic characteristics, and the VAS score between the groups. Chi-square tests were used to see the association between household size and insurance status. We used STATA version 13.0 (StataCorp. LLC, College Station, TX, USA) for conducting statistical analysis and adopted *p*-value of 0.05 or below as the statistically significant level.

### 2.6. Econometric Analysis

Multiple logistic regression models were used to predict the association between CBHI scheme enrolment and health status. We included five dimensions from the EQ-5D instrument and estimated scores as explanatory variables in separate models since these variables are moderately correlated (variance inflation factor from 1 to 3). The CBHI scheme enrollment was the dependent variable in each model. Demographic and household characteristics (e.g., age, gender, and household size) were included as control variables in each model. The models were specified as follows,

logit(Y_i_) = β_1_X_1i_ + β_2_X_2i_ + β_3_X_3i_ + … + ε_i_ (1)

where, Y_i_ = CBHI enrollment (1 = yes, 0 = No), X_1_, X_2_, X_3_ ... are explanatory variables, β_1_, β_2_, β_3_ ... represent the estimated coefficients and ε_i_ is the random error term of the model. The adjusted odds ratios were estimated as an exponential of the coefficient. The 95% confidence intervals of adjusted odds ratios are presented.

## 3. Results

Table 2 presents the demographic and socioeconomic characteristics of the respondents. The results showed that most of the participants were an adult (aged less than 60 years) in both insured (95.8%) and uninsured (95.5%) group. In the insured group, 51.7% were female and 48.3% were male and in the uninsured group 56.0% were female and 44.0% were male. Household size was larger in the insured group (52.6% had 6 or more members) compared to the uninsured group (30.3% had 6 or more members). There was no significant difference in the background characteristics between the insured and uninsured group respondents other than the household size (Table 2).

**Figure 1:**
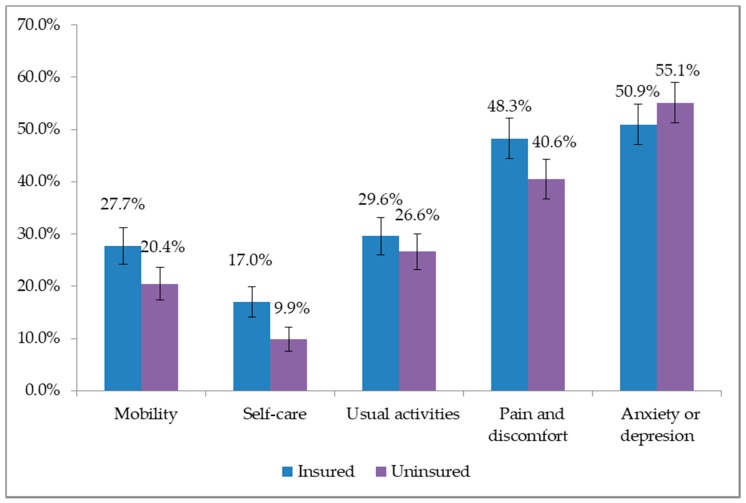
Proportion reported problem in EQ-5D dimensions between insured and uninsured.

Of the insured respondents, 50.9%, 48.3%, 29.6%, 27.7% and 17.0% reported having problem of “anxiety or depression”, “pain and discomfort”, “usual activities”, “mobility” and “self-care” respectively (Figure 1). However, a significant difference was found between the insured and uninsured population in case of “mobility” (*p* = 0.002), “self-care” (*p* = 0.000), “pain and discomfort” (*p* = 0.005).

Table 3 reports the health status and self-reported illness in terms of EQ-5D dimensions among the insured and uninsured respondents. The mean EQ-5D score was significantly different (*p* = 0.003) between the two groups. The mean VAS score for the uninsured group (77.3; 95% CI = 75.9–78.7) was higher than that of the insured group (77.0; 95% CI = 75.5–78.5) although the difference was not statistically significant.

The logistic regression analysis showed the association between the CBHI enrollment and dimensions of the EQ-5D (Table 4). We found that individuals having problems in “mobility” (OR = 1.65; 95% CI: 1.25–2.17), “self-care” (OR = 2.29; 95% CI: 1.62–3.25) and “pain and discomfort” (OR = 1.43; 95% CI: 1.13–1.81) were more likely to enroll in the CBHI scheme than individuals having no such problems. The households with 4 to 5 members and more than 6 members were significantly more likely to enroll in the CBHI scheme compared to the households with fewer than 4 members in all five models.

In model 6, CBHI scheme enrollment was regressed with the EQ-5D scores of individuals along with other background characteristics (Table 5). The odds ratio for the EQ-5D score is 0.46 (95% CI: 0.31, 0.69). This implies individuals with higher EQ-5D scores (a higher score refers to better health) were less likely to enroll in CBHI scheme.

## 4. Discussion

We have observed adverse selection among enrollees of a voluntary CBHI scheme when assessing the association between health status and enrolment. Our descriptive analysis showed that the EQ-5D health status of the insured individuals was significantly lower than matched uninsured individuals. When adjusting for confounding factors with multiple regression models, we found that the individuals with higher EQ-5D scores were about half as likely to enroll in CBHI scheme. It can thus be argued that adverse selection was present in the CBHI scheme. Such a problem can be a threat to the financial sustainability of the scheme. Our findings were similar to a previous study conducted in China, which found the presence of adverse selection in the Rural Mutual Health Care scheme [14]. Unlike our study, the Chinese study applied two questions for assessing the health status of the individuals, which included whether individuals suffered from any illness in the last month before the survey and the severity of that illness. A study in Burkina Faso, however, did not find any association between the health status of the household and CBHI enrollment [18].

We analyzed the relationship between insurance enrolment and five dimensions of health condition, including the physical and mental health status of individuals captured in the EQ-5D instrument. Among the specific health dimensions, three of them (“mobility”, “self-care”, and “pain and discomfort”) had significant association with the enrolment in CBHI scheme. Between the two other health dimensions (“usual activities” and “anxiety or depression”), “usual activities” was not significantly associated with the enrolment to CBHI scheme although it was at the borderline of significance. The odds-ratio of “usual activities” was 1.24 (95% CI: 0.96–1.61), which could be considered as an indication of higher enrolment of people with a problem in “usual activities”. Further, the “anxiety or depression” dimension was not significantly associated with “insurance enrolment”. In the context of the CBHI scheme analyzed here, the mental health services were not included in the benefits package and it is possible that it had not been reflected in the enrolment status of the individuals into the scheme.

Several solutions have been undertaken by health policymakers and private health insurance providers to reduce adverse selection in health insurance schemes. For instance, (i) mandatory enrollment of all individuals in the catchment area of the scheme; (ii) increasing the waiting period for accessing healthcare after enrolment in the scheme; (iii) introducing experience ratings or setting different premiums for different risk groups; (iv) cherry picking members or enrolling only low-risk people [14,24]. Many of these approaches may not be appropriate in the context of informal workers in Bangladesh. In general, CBHI schemes are subject to voluntary enrolment and the first remedy, mentioned above, e.g., compulsory enrolment, could not be considered since in the context of this current CBHI scheme, it is likely that the informal workers do not have a regular and steady income. Therefore, it was not imperative to enroll all informal workers and their household members in the scheme. From the beginning of the scheme, a 2-week waiting period for out-patient care and a 4-week period for inpatient care benefits were introduced. These waiting periods might have some effect on reducing the adverse selection. While more extended waiting periods could be a solution for lowering adverse selection, it might appear as a discouraging factor for potential enrollees to join a voluntary insurance scheme.

Introduction of experience ratings also may not be an appropriate way of reducing adverse selection in the context of this CBHI scheme since administering and overseeing the medical condition of potential enrollees would be very costly and might lead to financial challenges for sustainability. Since the income and health risk of potential scheme enrollees were difficult to monitor, the community-rating of the premium might be an appropriate solution for CBHI schemes targeting low-income informal workers. It should be noted here that the fourth way of reducing adverse selection, e.g., cherry picking or ensuring only the low-risk people was inappropriate since the scheme targeted the informal sector workers and their household members and any biases towards low-risk people would result in further inequities.

The conventional remedies of adverse selection appeared to not be applied often in the context of CBHI scheme [9]. In such a situation, we found that enlargement of the pool of members to allow healthy-to-ill cross-subsidization within the scheme enrollees would be a possible way to tackle the financial challenges of this CBHI scheme. However, it might be challenging to accumulate a large pool of members under the CBHI scheme. Khan and Ahmed found in an earlier study that the educational intervention to the informal workers could be a useful tool for increasing willingness to join a CBHI scheme and enlarge the coverage of the scheme [25].

A study conducted in China reported that adverse selection was relatively higher in an insurance scheme when households were partially enrolled (enrolled a few members) compared to fully enrolled (enrolled all members) as the partially enrolled households chose insurance for the members who had worse health status among them [14]. Several studies conducted in Sub-Saharan African settings reported that the obligation to enroll the entire household could reduce adverse selection [26,27]. This might decrease the possibility of enrolling only sick members of the households in the scheme. However, in our study, the benefits package included coverage of up to 6 members of the households with a flat-rate premium and coverage of more household members with an additional premium. Since the average household size of Bangladesh and the study sample were 4.5, our current scheme should have reduced the adverse selection seen with partial household enrolment.

One limitation of the current study was that the EQ-5D information was not collected for all members of households in the insured and uninsured groups for assessing the association between insurance enrollment and health status. However, we collected information from the selected adult wage-earning member of the households who might have influenced the decision making of insurance enrolment of the household. Another limitation is that the findings of this study could not be generalized to the whole country since this study only included a pilot intervention in a single district of Bangladesh.

## 5. Conclusions

This study showed the evidence of adverse selection in a CBHI scheme for informal workers in Bangladesh. The government of Bangladesh adopted the Health Care Financing Strategy 2012–2032 as a plan for achieving Universal Health Coverage where CBHI schemes were considered for securing financial protection of informal sector workers and their household members [8]. Adverse selection can be a challenge for achieving the success with CBHI schemes and consequently, achieving Universal Health Coverage. Adverse selection should thus be considered while designing and scaling up CBHI schemes in Bangladesh and in similar country settings.

## Figures and Tables

**Table 1:** The benefits package of the CBHI scheme.

Services	Co-Payment/Description *
Health benefits	
General practitioner (GP) Consultation	30 BDT (Market price = 300 BDT)
Medicine	20% discount from maximum retail price
Diagnostic tests	50% discount on market price
Specialist doctor consultation	100 BDT (Market price = 500 BDT)
Hospitalization	Maximum 4000 BDT per household per year
Periodic satellite clinics in remote rural areas	Free of charge
Non-health benefits	
Savings opportunity	▪ Each member/household could save minimum 10 BDT and maximum 100 BDT per week per household
Training programs	▪ 3 months computer training for student member of the household with a cost 1200 BDT (market price = 4500 BDT) ▪ 6 months sewing training for female workers (free of charge)

* 1 USD = 77.72 BDT [19].

**Table 2:** Background characteristics.

Characteristics	Insured (*N* = 646)	Uninsured (*N* = 646)	*p*-Value
	% (95% CI)	% (95% CI)	
Age group			
Adult (<60)	95.8 (93.9–97.1)	95.5 (93.6–96.9)	0.780 ^a^
Elderly (60+)	4.3 (2.9–6.0)	4.5 (3.1–6.4)	
Gender			
Female	51.7 (47.8–55.5)	56.0 (52.2–59.8)	0.120 ^a^
Male	48.3 (44.5–52.2)	44.0 (40.2–47.8)	
Household size			
Fewer than 4 members	6.8 (5.1–9.0)	15.8 (13.2–18.8)	0.001 ^b^
4–5 members	40.6 (36.8–44.4)	53.9 (50.0–57.7)	
6 members or more	52.6 (48.8–56.5)	30.3 (26.9–34.0)	

**^a^** *t*-test of proportion; ^b^ Chi-square test.

**Table 3:** Health status (EQ-5D and VAS score) and self-reported illness between insured and uninsured.

Characteristics	Insured	Uninsured	*p*-Value
	% (95% CI)	% (95% CI)	
EQ-5D mean score	0.704 (0.682–0.727)	0.749 (0.730–0.769)	0.003
EQ-5D median score	0.726	0.766	
VAS (mean score)	77.0 (75.5–78.5)	77.3 (75.9–78.7)	0.783
VAS (Median score)	80.0	80.0	
Self-reported chronic illness/symptoms	9.8 (7.5–12.0)	7.6 (5.5–9.6)	0.166

**Table 4:** Association between EQ-5D dimensions and CBHI enrollment.

Variables	Description	Model 1	Model 2	Model 3	Model 4	Model 5
Age group	Elderly, 60+ (Ref = Adult, <60)	0.810 (0.455–1.440)	0.891 (0.504–1.575)	0.910 (0.516–1.603)	0.887 (0.503–1.563)	0.980 (0.555–1.728)
Gender	Female (Ref = Male)	1.217 (0.965–1.534)	1.247 (0.989–1.574)	1.172 (0.931–1.476)	1.188 (0.944–1.495)	1.123 (0.893–1.411)
Household size	4–5 members (Ref = Fewer than 4 members)	1.747 ** (1.180–2.585)	1.793 ** (1.207–2.662)	1.730 ** (1.171–2.557)	1.713 ** (1.158–2.532)	1.719 ** (1.164–2.539)
	6 members or more (Ref = Fewer than 4 members)	4.034 *** (2.705–6.016)	4.272 *** (2.853–6.398)	3.982 *** (2.675–5.927)	3.957 *** (2.657–5.893)	3.913 *** (2.631–5.820)
Chronic illness	Yes (Ref = No)	1.218 (0.808–1.834)	1.252 (0.831–1.887)	1.231 (0.817–1.856)	1.178 (0.781–1.777)	1.332 (0.886–2.001)
Mobility	Any problem (Ref = No problem)	1.649 *** (1.253–2.170)	-	-	-	-
Self-care	Any problem (Ref = No problem)	-	2.290 *** (1.617–3.245)	-	-	-
Usual activities	Any problem (Ref = No problem)	-	-	1.244 (0.960–1.613)	-	-
Pain and discomfort	Any problem (Ref = No problem)	-	-	-	1.431 ** (1.133–1.807)	-
Anxiety or depression	Any problem (Ref = No problem)	-	-	-	-	0.878 (0.698–1.103)
Constant		0.347 *** (0.236–0.509)	0.331 *** (0.225–0.487)	0.375 *** (0.256–0.550)	0.343 *** (0.232–0.505)	0.436 *** (0.295–0.646)
*N*		1291	1291	1291	1291	1291
LR chi2(27)		90.12	99.84	79.94	86.32	78.45
Prob. > chi2		0.000	0.000	0.000	0.000	0.000
Pseudo R2		0.050	0.056	0.045	0.048	0.044

** *p* < 0.01, *** *p* < 0.001.

**Table 5:** Association between EQ-5D score and CBHI enrollment.

Variables	Description	Model 6 (Dependent = CBHI Enrollment; 1 = Insured, 0 = Uninsured) Odds Ratio (95% CI)
Age group	Elderly, 60+ (Ref = Adult, <60)	0.861 (0.488–1.518)
Gender	Female (Ref = Male)	1.196 (0.950–1.506)
Household size	4–5 members (Ref ≤ 4 members)	1.736 ** (1.173–2.570)
	≥6 members (Ref ≤ 4 members)	4.049 *** (2.715–6.040)
Chronic illness	Yes (Ref = No)	1.164 (0.771–1.757)
Eq-5D score		0.460 *** (0.307–0.689)
Constant		0.711 (0.444–1.137)
*N*		1291
LR chi2(27)		−848.9
Prob. > chi2		0.000
Pseudo R2		0.051

** *p* < 0.01, *** *p* < 0.001.
